# 3-Deoxysappanchalcone isolated from *Caesalpinia sinensis* shows anticancer effects on HeLa and PC3 cell lines: invasion, migration, cell cycle arrest, and signaling pathway

**DOI:** 10.1016/j.heliyon.2022.e11013

**Published:** 2022-10-12

**Authors:** Dian Lv, Qi Lai, Qi Zhang, Ji-hong Wang, Yuan-ce Li, Guang-Zhi Zeng, Jun-Lin Yin

**Affiliations:** Key Laboratory of Chemistry in Ethnic Medicinal Resources, State Ethnic Affairs Commission & Ministry of Education; School of Ethnic Medicine, Yunnan Minzu University, Kunming, China

**Keywords:** *Caesalpinia sinensis*, Proliferation, Cell cycle, Migration, Invasion

## Abstract

To study the antitumor activity of compound 3-desoxysulforaphane (3-DSC) isolated from *Caesalpinia sinensis*, SRB assay, clone formation assay, flow cytometric cell cycle assay, scratch assay, transwell assay, and molecular docking were used to investigate the inhibitory effect of 3-DSC on HeLa and PC3 cells. The results showed that 3-DSC inhibited the cell migration and invasion by down-regulating expression of N-cadherin, Vimentin, MMP-2, and MMP-9 in HeLa and PC3 cells; It also inhibits cell proliferation by promoting the expression of CDK1 (cyclin-dependent kinases 1) and CDK2 (cyclin-dependent kinases 2), which arrests the tumor cell cycle at G2 phase. 3-DSC inhibits phosphorylation of AKT and ERK and upregulates the expression of the tumor suppressor gene p53. Molecular docking results confirmed that 3-DSC could bind firmly to AKT. In conclusion, 3-DSC inhibited the proliferation, migration and invasion of HeLa and PC3 cells.

## 1 Introduction

Malignant tumor is a serious disease that endangers human life and health [1]. Cervical cancer is one of the leading causes of death in women worldwide, with approximately 530,000 new cases and 275,000 deaths each year [2, 3]. Prostate cancer is one of the most common malignant tumors in male medicine [4], and in China, the incidence of prostate cancer is showing a year-on-year increase [2]. Currently, surgery and radiotherapy are the main treatments, but most patients still suffer from recurrence and metastasis [5]. Therefore, the search for effective therapeutic agents for cervical and prostate cancers is crucial.

The search for effective anti-tumor drugs from plants has become a hot research topic for pharmacological researchers around the world with a broad prospect. Such as paclitaxel [6], betulinic acid [7], triterpenoid [8], and artemisinin [9] derivatives are attracting more and more attention from pharmacologists due to their unique structure and good anti-tumor activity. Previous studies showed that a diet rich in flavonoids will reduce the risk of colon, prostate, and breast cancers [10]. *Caesalpinia sinensis* contains flavonoids, terpenoids, alkaloids, and steroids with anti-inflammatory, antioxidant, antiviral, antibacterial, and neuroprotective activities [11, 12]. 3-deoxysulforaphane (3-DSC) is the active ingredient in *C. sinensis* [13]. Meanwhile, 3-DSC activates AKT (serine/threonine-specific protein kinase)/mTOR to induce HO-1 for anti-inflammation and also inhibits intracellular CCL5 and CXCL10 secretion to protect host cells from influenza virus-induced inflammatory, with anti-influenza effects [14, 15]. In addition, 3-DSC has a hair growth effect by regulating the growth of hair follicle dermal papilla cells through the WNT/β-catenin and STAT signaling pathways [14]. Regarding antitumor, 3-DSC inhibits colon cancer cell proliferation and induces G2 phase arrest and proliferation [16]. However, there are few reports of 3-DSC inhibiting the proliferation and mechanism of cervical and prostate cancer.

In this study, the compound 3-DSC was isolated from *C. sinensis*, and the cell lines HeLa and PC3 sensitive to 3-DSC were screened. The inhibitory effect of 3-DSC on HeLa and PC3 was verified by cell cycle arrest, migration and invasion, and cell viability assays. The mechanism of 3-DSC inhibition of HeLa and PC3 was probed by molecular docking and western blot.

## 2 Materials and methods

### 2.1 Isolation and identification of 3-DSC

The dried branches and leaves of *C. sinensis* (10 kg) were extracted with methanol to obtain 1.1 kg of infusion. *C. sinensis* infusion was mixed in 5 L water and extracted with n-butanol to obtain a 50.12 g extract. The extract was first subjected to column chromatography on silica gel (dichloromethane; methanol, 250:1) and then purified by high-performance thin-layer chromatography (HPTLC) to get a pale yellow solid of 8.2 mg (3-DSC) [13]. NMR data (BrukerAV, Germ): ESI-MS *m*/*z*: 269.2 [M−H]^−^, ^1^HNMR (400 MHz, CD_3_OD) *δ*: 7.65 (1H, d, *J* = 15.7 Hz, H-*β*), 7.40 (1H, d, *J* = 15.7 Hz, H-*α*), 7.57 (1H, d, *J* = 8.5 Hz, H-6′), 6.45 (1H, dd, *J* = 8.5, 2.0 Hz, H-5′), 6.50(1H, d, *J* = 2.0 Hz, H-3′), 7.49 (2H, d, *J* = 8.7 Hz, H-2, 6), 6.82 (2H, d, *J* = 8.7 Hz, H-3, 5), 3.83 (3H, s, –OCH_3_). ^13^C-NMR *δ*: 188.95 (C=O), 162.68 (C-2′), 160.49 (C-4′), 159.72 (C-4), 141.46 (C-*β*), 132.33 (C-6′), 130.35 (C-2, 6), 126.09 (C-1), 124.00 (C-*α*), 120.32 (C-1′), 115.96 (C-3, 5), 107.94 (C-5′), 99.33 (C-3′), 55.74 (-OCH_3_). The ^13^C-NMR data were identified as 3-deoxysappanchalcone [13].

### 2.2 Cell culture

HeLa was cultured in a DMEM (BI, Israel) medium containing 10% FBS (BI, Israel) and PC3 was cultured in RPMI 1640 (BI, Israel) medium containing 10% FBS in a cell culture incubator (Thermo Scientific, USA) at 37 °C with 5% CO_2_. Depending on cell growth and cell status, cells were passaged approximately once every 3 days to maintain good cell growth. All cell plates were purchased from NEST Biotechnology (Wuxi, China).

### 2.3 Cell proliferation and death patterns experiments

HeLa and PC3 cells at the logarithmic growth stage were inoculated at 5 × 103 cells/well in 96-well plates. 3-DSC was incubated with HeLa and PC3 cells at the concentrations of 0, 5, 10, 20, and 50 μM for 48 h, and IC_50_ (half maximal inhibitory concentration) was calculated. Similarly, 20 μM of 3-DSC combined with 1 μM apoptosis inhibitor Z-VAD-FMK (MCE, USA), 1 μM autophagy inhibitor HMY1485 (MCE, USA), 1 μM ferroptosis inhibitor Fer-1 (MCE, USA), 5 μM ferroptosis inhibitor Lip-1 (MCE, USA), and 1 μM necrosis inhibitor Nec-1 (MCE, USA) to explore the cell proliferation and death patterns induced by 3-DSC in HeLa and PC3 cells. The cells were fixed in 50% TCA for 1 h and the 96-well plates were air-dried. 4 mM Tris solution was used to lyse the substrate and the absorbance was measured at 515 nm with a microplate reader, and the cell viability was calculated from the results [17].

### 2.4 Colony formation assay

HeLa and PC3 cells at the logarithmic growth phase were spread in 12-well plates (200 cells/well) and incubated in a cell culture incubator at 37 °C with 5% CO_2_ for about 3 d. When cell mass formed, 3-DSC was incubated for 10 d at 0, 5, 10, and 20 μM. After completing the experiment, anhydrous ethanol was used to fix HeLa and PC3 cells for 15 min, 0.4% crystalline violet (Solarbio, China) was stained for 5 min, observed under a microscope, and photographed with a camera.

### 2.5 Wound healing assay

HeLa and PC3 cells at the logarithmic growth stage were taken at 2 × 10^5^ cells/well, spread in 12-well plates, and incubated for 24 h at 37 °C in a cell culture incubator with 5% CO_2_. When the cells grew all over the bottom of the dish and were in a monolayer, drawn a uniform vertical line with the pipette tip and removed the suspended cells, taken photos, and recorded. Next, cells were incubated with 0, 5, 10, 20 μM of 3-DSC for 48 h. Photographs were taken at the same locations and the migration rate was calculated using Image J software from the photographs recorded at 0 h and 48 h.

### 2.6 Transwell migration and invasion assay

As for invasion, transwell upper chamber porous polycarbonate membrane filter membranes (NEST Biotechnology, China) were gelled with 50 μL of artificial MATRIGEL (Corning, USA) and gelled flat at 37°C for 2–3 h. HeLa and PC3 cells in the upper chamber were inoculated with serum-free medium (200 μL) at 1 × 105 cells/well, while the lower chamber medium (600 μL) containing 10% serum and 0, 5, 10, 20 μM 3-DSC was incubated for 24 h at 37°C in a 5% CO_2_ incubator. 4% crystalline violet stained for 5 min, PBS washed; cotton swabs were used to wipe the upper cells of the small chamber and the lower chamber was photographed and recorded. Experiments without the use of artificial MATRIGEL were transwell migration.

### 2.7 Cell cycle assay

HeLa and PC3 cells at the logarithmic growth stage were plated in 12-well plates at 1.2 × 10^5^ cells/well and incubated for 24 h at 37°C in a 5% CO_2_ incubator. Next, HeLa and PC3 cells were incubated for 24 h with 3-DSC (5, 10, 20, 50 μM) and the positive drug Paclitaxel (PTX, 0.5 μM). After completion of the experiment, cells were collected, fixed overnight in 70% ethanol, stained with PI (propidium iodide, Yeasen, China) and detected by flow cytometry [18, 19].

### 2.8 Western blot assay

HeLa and PC3 cells were collected and RIPA lysate was used to lyse the cells on ice to extract the total protein. Each protein was sampled in 10% SDS-PAGE for electrophoresis, wet transferred onto PVDF membranes, and 5% skimmed milk powder closed at room temperature for 2 h. 2% skimmed milk powder dilutions of primary antibodies N-cadherin (1:5000, Abcam, UK), Vimentin (1:5000, Abcam, UK), MMP-2 (1:3500, Abcam, UK), MMP-9 (1:3500, Abcam, UK), p-Erk (1:3000, Abcam, UK), CDK1 (1:3000, Abcam, UK), CDK2 (1:4000, Abcam, UK), p53 (1:3000, Abcam, UK), Akt (1:3000, Abcam, UK) and β-actin (1:15,000, Abcam, UK) were incubated overnight at 4 °C and the membranes were washed with TBST. The secondary antibodies were diluted in 2% skimmed milk powder (1:7000) and incubated for 2 h at room temperature, and the membrane was washed with TBST. Finally, the ECL chemiluminescent reaction solution is developed and the protein is detected on the chemiluminescent developer, along with semi-quantitative greyscale analysis [20, 21]. All raw data of western blot protein bands were displayed in supplementary figures.

### 2.9 Molecular docking assay

The AKT (PDB ID: 1H10) protein crystal structure was downloaded from the database (http://www.rcsb.org) in PDB format. After removing extraneous small molecules from the protein molecules using Pymol 2.2 software, the protein molecules were imported into AutoDock Tools-1.5.6 software to remove water molecules and add hydrogen atoms before being saved as pdbqt files. Next, 3-DSC was imported into AutoDock Tools-1.5.6 software for molecular docking with the ligand-free AKT protein. The molecular docking results were visualized using Pymol 2.2 software and Discovery Studio 2019 software.

### 2.10 Statistical analysis

Statistical analysis was performed using GraphPad Prism 8 software, with three independent replications of the experiment, expressed as mean ($\bar{x}$) ± standard deviation (SD), and a one-way ANOVA test was used to analyze significant differences between groups.

## 3 Results

### 3.1 3-DSC inhibits the proliferation of HeLa and PC3 cells

**Figure 1:**
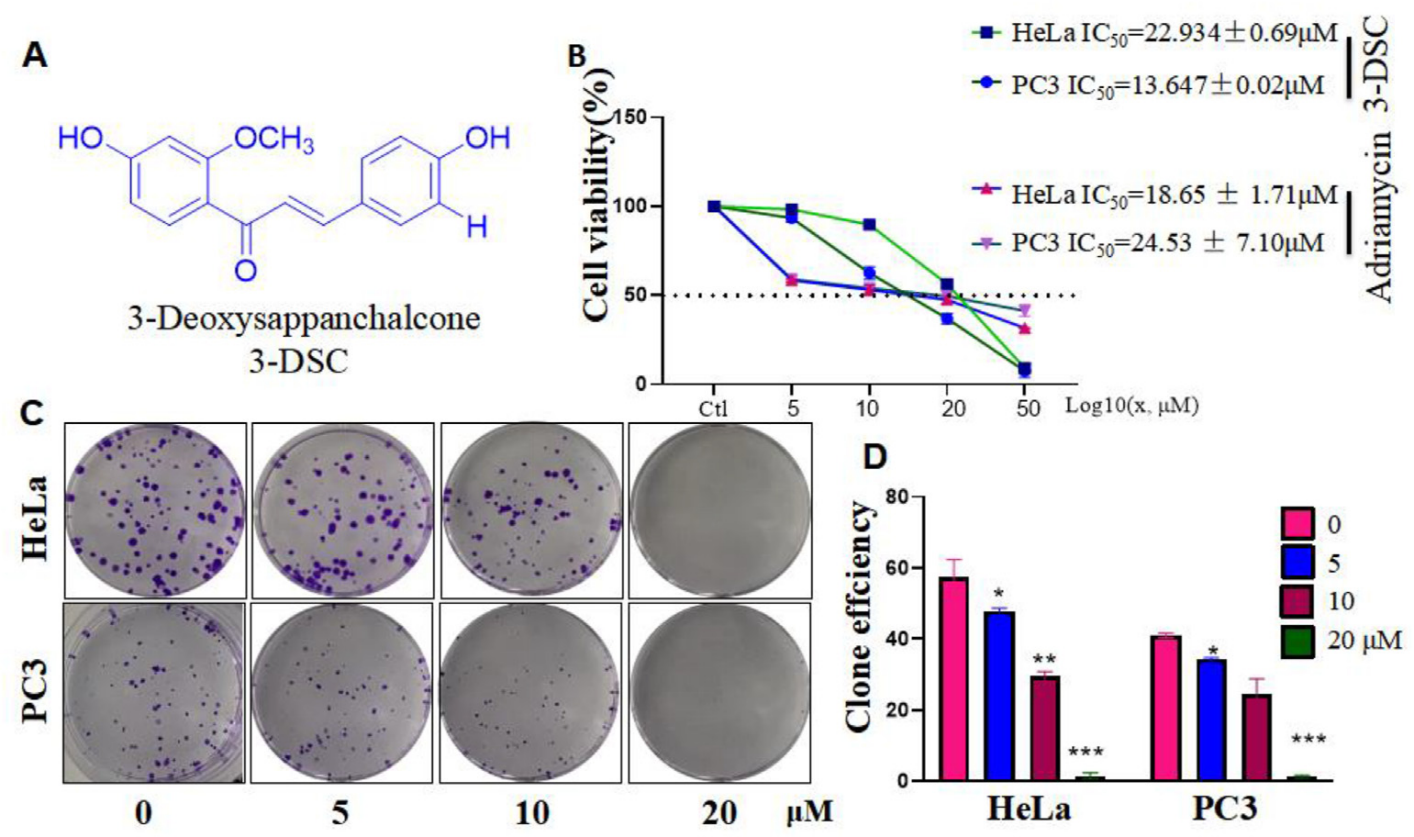
Effect of 3-DSC on cell proliferation in HeLa and PC3 cells. A: Chemical structure of 3-DSC. B: SRB cell viability assay. C and D: Clone formation assay. ∗ indicates comparison with 3-DSC free group (0 group), ∗p < 0.05, ∗∗p < 0.01,∗∗∗p < 0.001.

3-DSC is a natural flavonoid with 2 benzene rings and the structural formula is shown in Figure 1A. The SRB assay yielded that 5, 10, 20, and 50 μM of 3-DSC inhibited HeLa and PC3 in a dose-dependent manner, with an IC_50_ of 22.934 ± 0.69 μM for HeLa and 13.647 ± 0.02 μM for PC3. And, IC_50_ of adriamycin on HeLa is 18.65 ± 1.71 μM, for PC3 is 24.53 ± 7.10 μM (Figure 1B). Meanwhile, clone formation assays verified the effect of 3-DSC on the proliferative capacity of HeLa and PC3. The rate of clone formation in HeLa and PC3 decreased with increasing concentrations of 3-DSC in cells, with almost no clone formation at 20 μM (Figure 1C and D). Taken together, 3-DSC has an inhibitory value-added and death-inducing effect on HeLa and PC3.

### 3.2 3-DSC inhibits migration and invasion of HeLa and PC3 cells

**Figure 2:**
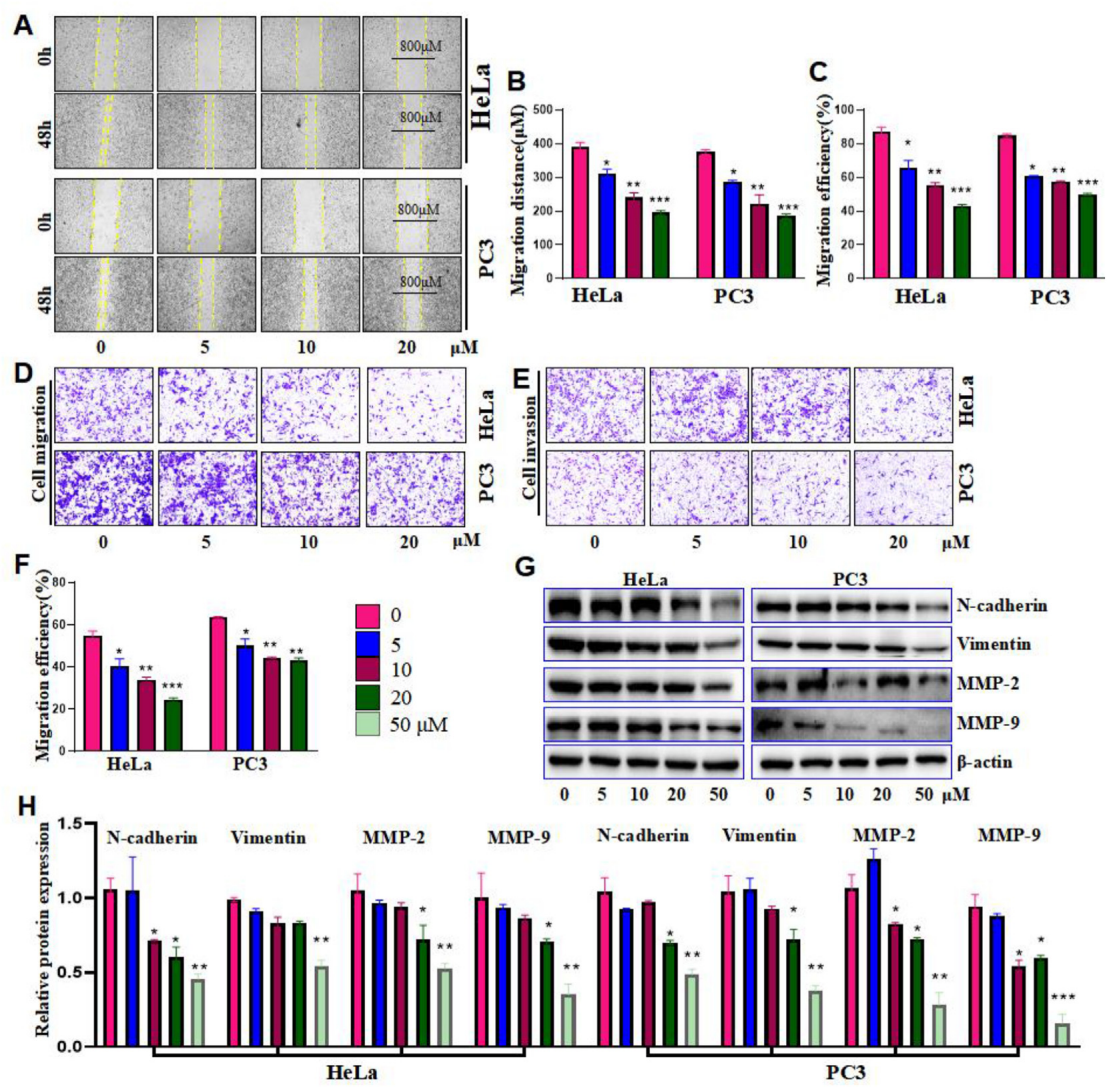
Effect of 3-DSC on migration and invasio in HeLa and PC3 cells. A and B: wound Healing Assay. C and D: Transwell migration. E and F: Transwel invasion. G and H: The proteins expression of N-cadherin, Vimentin, MMP-2, and MMP-9. ∗ indicates comparison with 3-DSC free group (0 group), ∗*p* < 0.05, ∗∗*p* < 0.01, ∗∗∗*p* < 0.001.

The ability of cells migration and invasion were often used as an indicator of the strength of the tumor cells affected. The migration distances of HeLa and PC3 cells were measured by wound healing assay at 0 h and 48 h, respectively. As shown in Figure 2A and B, compared with the 3-DSC free group, 5, 10, and 20 μM of 3-DSC significantly inhibited the migration of both HeLa and PC3 cells in a dose-dependent manner. Next, transwell cell migration and invasion assays were performed. Compared with the 3-DSC free group, the migratory capacity of HeLa and PC3 was reduced in a 3-DSC concentration-dependent manner (Figure 2C and D). Similarly, compared with the 3-DSC free group, the invasive ability of HeLa and PC3 was reduced in a 3-DSC concentration-dependent form (Figure 2E and F). To further explore the migration and invasion mechanisms of HeLa and PC3, western blot was performed to detect N-cadherin proteins that maintain cell morphology, Vimentin proteins that make up the cytoskeleton, and MMPs (matrix metallo proteinases), extracellular matrix (ECM) degrading enzymes. As shown in Figure 2G and H, 5, 10, 20 and 50 μM of 3-DSC inhibited the expression of N-cadherin, Vimentin, MMP-2, and MMP-9 in a dose-dependent manner compared to the 3-DSC free group. These results suggest that 3-DSC can inhibit the expression of N-cadherin, Vimentin, and MMPs, thereby inhibiting cell migration and invasion.

### 3.3 3-DSC induces cell cycle arrest in HeLa and PC3

**Figure 3:**
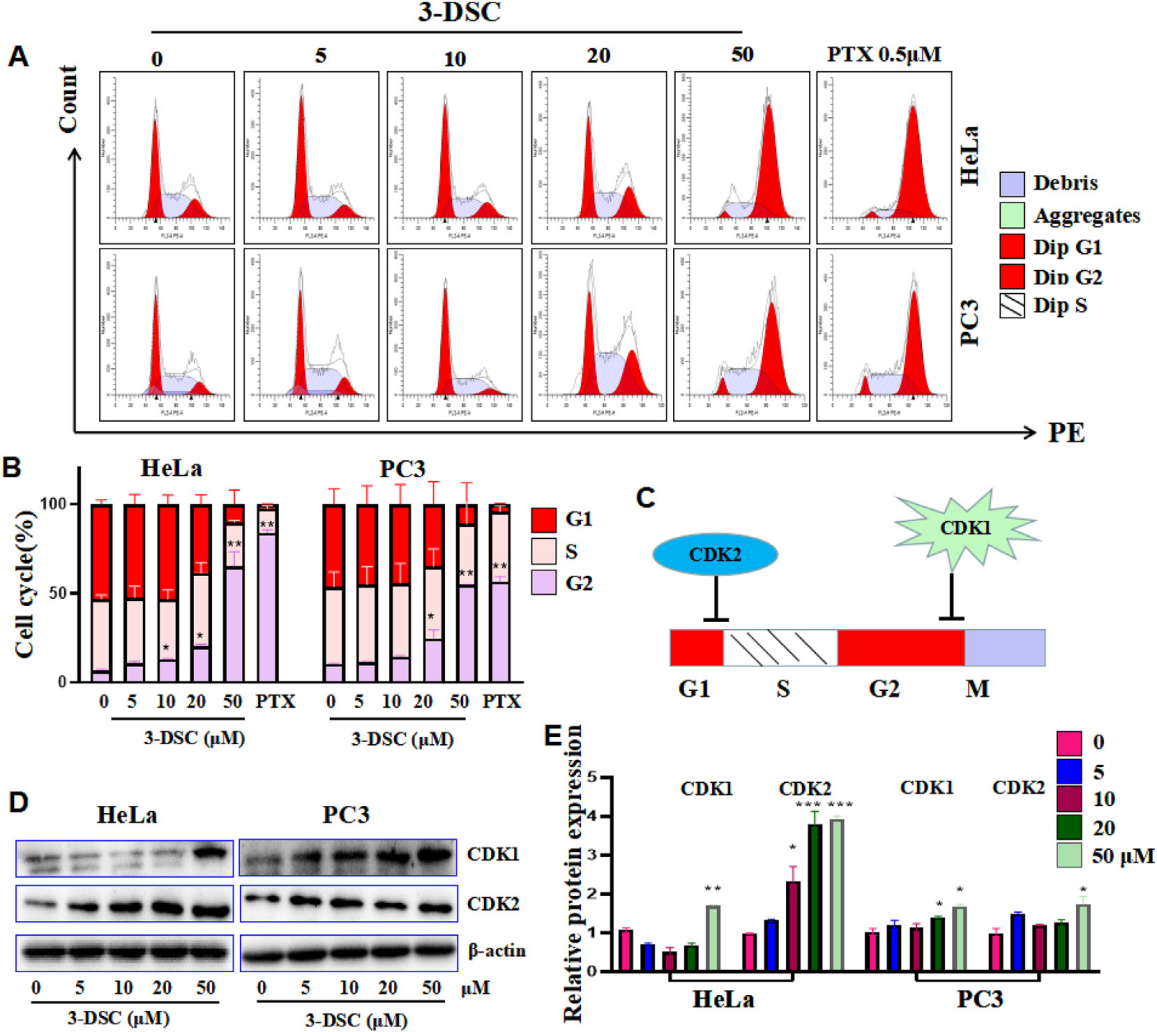
3-DSC effects on cell cycle. A and B: Cell cycle. C: Schematic representation of CDK1 and CDK2 regulation of cell cycle. D and E: The proteins expression of CDK1 and CDK2. ∗ indicates comparison with 3-DSC free group (0 group), ∗*p* < 0.05, ∗∗*p* < 0.01,∗∗∗*p* < 0.001.

Blocking the cell cycle is one of the mechanisms that inhibit the growth of tumor cells. The cell cycle was analyzed by flow cytometry and HeLa and PC3 cell cycles showed a decrease in the G1 phase and an increase in the G2 phase with a dose-dependent relationship when 3-DSC was applied for 24 h. Moreover, 50 μM of 3-DSC had about the same effect as 0.5 μM of the positive drug paclitaxel (Figure 3 A and B). CDK1 and CDK2 are closely associated with the cell cycle, with CDK1 acting as a G2-M monitoring point and CDK2 as a G1-S phase monitoring point (Figure 3C). Using Western Blot for changes in cycle-related proteins, expression of both CDK1 and CDK2 could be induced by 3-DSC in a dose-dependent manner (Figure 3D and E). The above results suggest that 3-DSC may block the replication of genetic material in G2 phase, thereby retarding the cell division and growth.

### 3.4 The death mode and mechanism of HeLa and PC3 inducted by 3-DSC

**Figure 4:**
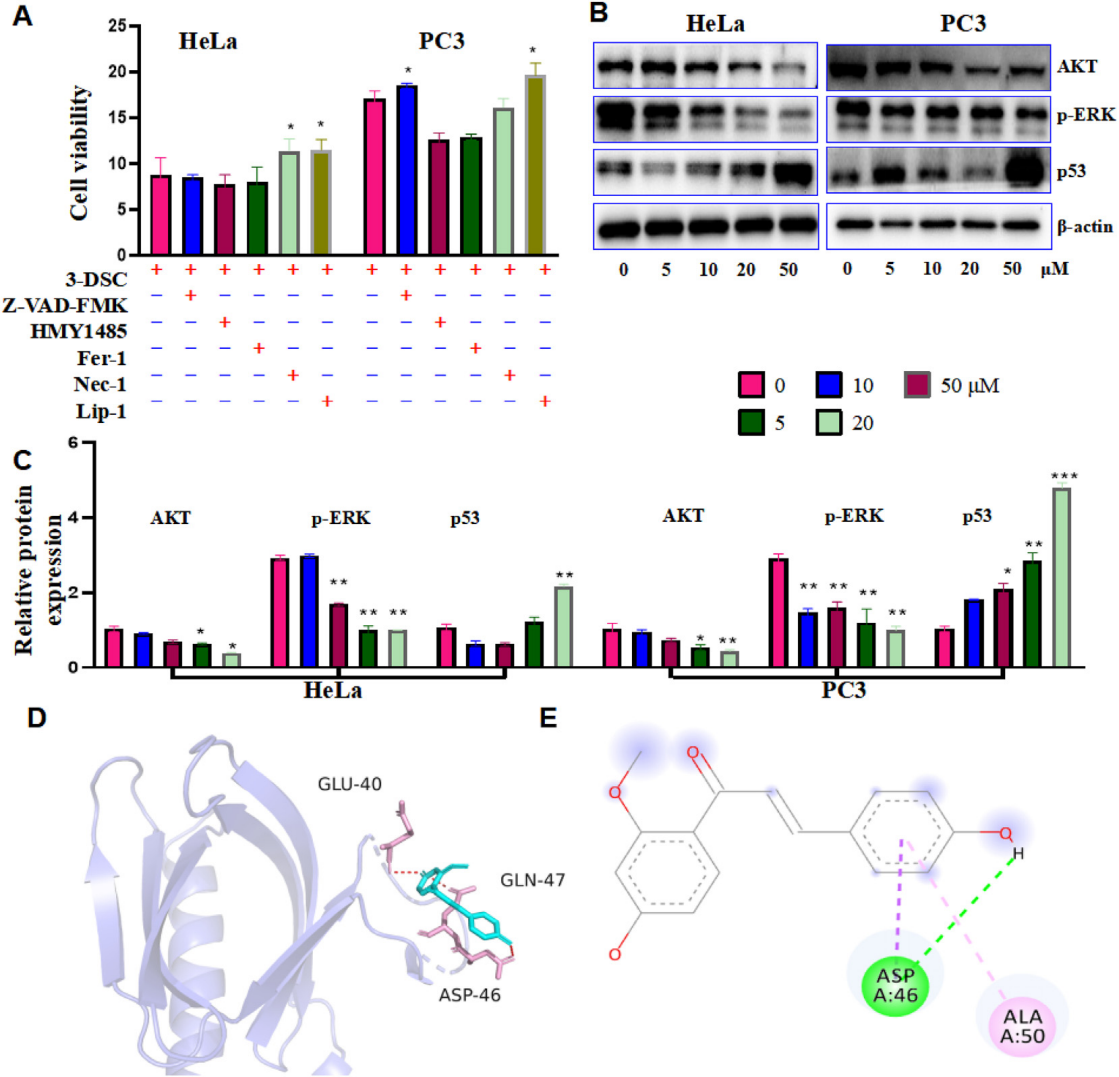
Effect of 3-DSC on the mode and mechanism of death of HeLa and PC3. A: SRB cell viability assay to detect the mode of death. B and C: The proteins expression of AKT, p-ERK and p53. D and E: Molecular docking results of 3-DSC with AKT. Z-VAD-FMK is a Pan-Caspase inhibitor; MHY1485 is a potent, cell-permeable mTOR agonist that also effectively inhibits autophagy; Fer-1 (Ferrostatin-1) is a potent and selective inhibitor of ferroptosis; Nec-1 (Necrostatin-1) is a specific RIP1 (RIPK1) inhibitor that inhibits cell necrosis; Lip-1 (Liproxstatin-1) is a potent inhibitor of ferroptosis. ∗ indicates comparison with 3-DSC free group (0 group), ∗p < 0.05, ∗∗p < 0.01, ∗∗∗p < 0.001.

To explore the way 3-DSC induces death in HeLa and PC3, an apoptosis inhibitor, an autophagy inhibitor, ferroptosis inhibitors, and a necrosis inhibitor were pretreated to protect HeLa and PC3 for 2 h and then induced with 20 μM of 3-DSC. Nec-1 and Lip-1 were able to inhibit 3-DSC-induced HeLa cell death, and Z-VAD-FMK and Lip-1 were able to inhibit 3-DSC-induced PC3 cell death (Figure 4A). Furthermore, as shown in figures 4B and C, western blot was performed to detect changes in the cell proliferation-related proteins AKT, p-ERK, and p53. AKT and p-ERK expression decreased with the increasing 3-DSC concentration. In contrast, p53 increased with the increasing 3-DSC concentration. To further investigate the effect of 3-DSC on the upstream protein AKT, molecular docking was carried out. As shown in Figure 4D and E, the binding energy of 3-DSC to AKT is much less than 0, suggesting that 3-DSC binds closely to AKT. GLU-40, ASP-46, and GLN-47 are the active sites of AKT proteins and bind to 3-DSC mainly by hydrogen and π bonds. The above experimental results suggest that 3-DSC may target AKT to regulate p-ERK and p53 and thus influence the mode of cell death.

## 4 Discussion

Cervical and prostate cancers are frequently occurring malignancies that pose a serious threat to people’s lives [1]. The continued development of medical treatment modalities such as surgery and radiotherapy has led to a reduction in the death rate from cancer [22]. However recurrence rates remain high and chemotherapy resistance remains unresolved [23]. Although targeted drugs have achieved good therapeutic results, the high cost of the drugs makes them unaffordable for many patients [24]. This study shows that 3-DSC exhibits good activity in vitro against tumor cells HeLa and PC3, and focuses on the mechanisms by which 3-DSC inhibits cell migration, invasion and induces cycle arrest.

In this study, 3-DSC significantly inhibited the viability of HeLa and PC3, with lower concentration IC_50_ for both HeLa (22.93 ± 0.96) μM and PC3 (13.647 ± 0.02) μM. Cell migration and invasion processes associated with Epithelial-Mesenchymal Transition (EMT) [25]. During EMT, tumor cells undergo a morphological transition from an epithelial phenotype to a mesenchymal phenotype, while acquiring greater invasive capacity [26]. N-cadherin plays a key role in maintaining cell morphology by maintaining the stability of connections between epithelial cells [27]. A decrease in N-cadherin is an important marker of EMT [28]. Vimentin is an essential component of the cytoskeleton, maintaining the integrity of the cytoskeleton, morphology, and bridging granules [27]. Vimentin is also associated with cell motility and is a marker of EMT [29]. MMPS is zinc-dependent endopeptidases that degrade essentially all extracellular matrix components, with MMP-2 and MMP-9 playing a role in the migration and invasion of cancer [30]. MMP-2 and MMP-9 catalyze the degradation of most extracellular matrix and basement membrane components and promote tumor angiogenesis, thereby enhancing tumor invasion and metastasis [31, 32]. In this experiment, the migratory and invasive capacities of the cells were reduced in the presence of 3-DSC, and N-cadherin, Vimentin, MMP-2, and MMP-9 were all reduced. These results suggested that 3-DSC may inhibit the metastasis and spread of tumor cells and thus provide better efficacy.

3-DSC induced cell death in HeLa and PC3, we can find that 3-DSC induces HeLa cells through necrosis and ferroptosis, and PC3 cells mainly through apoptosis and ferroptosis. In-depth mechanistic studies have shown that the molecular docking between 3-DSC and AKT is stable. AKT and p-ERK decreased by the action of 3-DSC, and the tumor suppressor protein p53 was highly expressed. The AKT signaling pathway is one of the most common signaling pathways in human cancers, regulating abnormal cell proliferation and a key aspect of tumor development [33, 34]. ERK1/2, a member of the mitogen-activated protein kinase superfamily, primarily mediates cell proliferation and division and has emerged as a target for intervention in cancer research [35, 36]. Previous studies have shown that inhibition of ERK phosphorylation can effectively inhibit cancer proliferation and metastasis during the development of breast cancer [37, 38, 39]. EMT has been shown to play a key role in tumourigenesis and invasive metastasis and is regulated by a variety of signaling pathways, including AKT and ERK [40]. Moreover, p53 is an important anti-cancer gene that causes apoptosis of cancer cells, thus preventing cancer [41]. Meanwhile, p53 has been reported to be involved in multiple modes of cell death [42, 43]. Overall, 3-DSC regulates the involvement of p-ERK and p53 in the cell death process and mode of death by targeting AKT.

Cell cycle regulation is critical for the body to maintain the orderliness of cell proliferation and the stability of genomic DNA, and its deregulation is central to tumorigenesis and is closely related to tumor development. Cyclin-dependent kinases (CDK) form complexes by phosphorylating cyclins, with the formation of the CDK4/6-Cyclin D1 complex playing a crucial role in G1 phase progression, the CDK2-Cyclin E1/A2 complex being critical in the late G1 to S phase, and the CDK1-Cyclin B1 complex acting mainly in G2 phase [44, 45]. p53 is an important oncogene and a key protein in the cycle detection pathway, and activation of p53 may lead to G1 or G2 phase block [44]. Studies have shown that p53 deficiency (mutation or deletion) induces G2/M phase block [46, 47, 48]. In this experiment, the G1 and S-phase ratios of HeLa and PC3 tumor cells were down-regulated and the G2 phase was increased under the effect of 3-DSC, while enhanced expression of CDK1 and CDK2 proteins were detected, and 3-DSC was also found to promote p53 protein expression in HeLa and PC3 cells. Taken together, these results suggest that 3-DSC induced G2 phase block in HeLa and PC3 cells and inhibited tumor cell proliferation.

## 5 Conclusion

In summary, in this study, the monomeric compound 3-DSC extracted from *C. sinensis* was investigated against cervical cancer HeLa and prostate cancer PC3. 3-DSC could target AKT to inhibit the migration and invasion of HeLa and PC3 through the ERK pathway and induce G2 blockage in HeLa and PC3 by modulating p53 to inhibit proliferation. Next, the monomeric compound 3-DSC will be enriched and the specific mechanism of its induction of cell death will be discussed in-depth and complemented with in vivo experiments.

## Declarations

### Author contribution statement

Dian Lv: Performed the experiments; Analyzed and interpreted the data.

Qi Lai: Performed the experiments; Analyzed and interpreted the data; Wrote the paper.

Qi zhang, Ji-hong Wang and Yuan-ce Li: Performed the experiments; Analyzed and interpreted the data; Contributed reagents, materials, analysis tools or data.

Guang-Zhi Zeng: Conceived and designed the experiments.

Jun-Lin Yin: Conceived and designed the experiments; Wrote the paper.

### Funding statement

This work was supported by the 10.13039/100014718National Natural Science Foundation of China (No. 21768005, 31760095, 81960639).

### Data availability statement

Data will be made available on request.

### Declaration of interest’s statement

The authors declare no conflict of interest.

### Additional information

No additional information is available for this paper.
