# Supplementary material for: 3-Deoxysappanchalcone isolated from Caesalpinia sinensis shows anticancer effects on HeLa and PC3 cell lines: invasion, migration, cell cycle arrest, and signaling pathway
Source: Heliyon. 2022 Oct 12;8(10):e11013. doi: 10.1016/j.heliyon.2022.e11013 (PMC9582709; doi:10.1016/j.heliyon.2022.e11013)
Supplement: supplementary figures [file mmc1.ppt]

## Slide 1
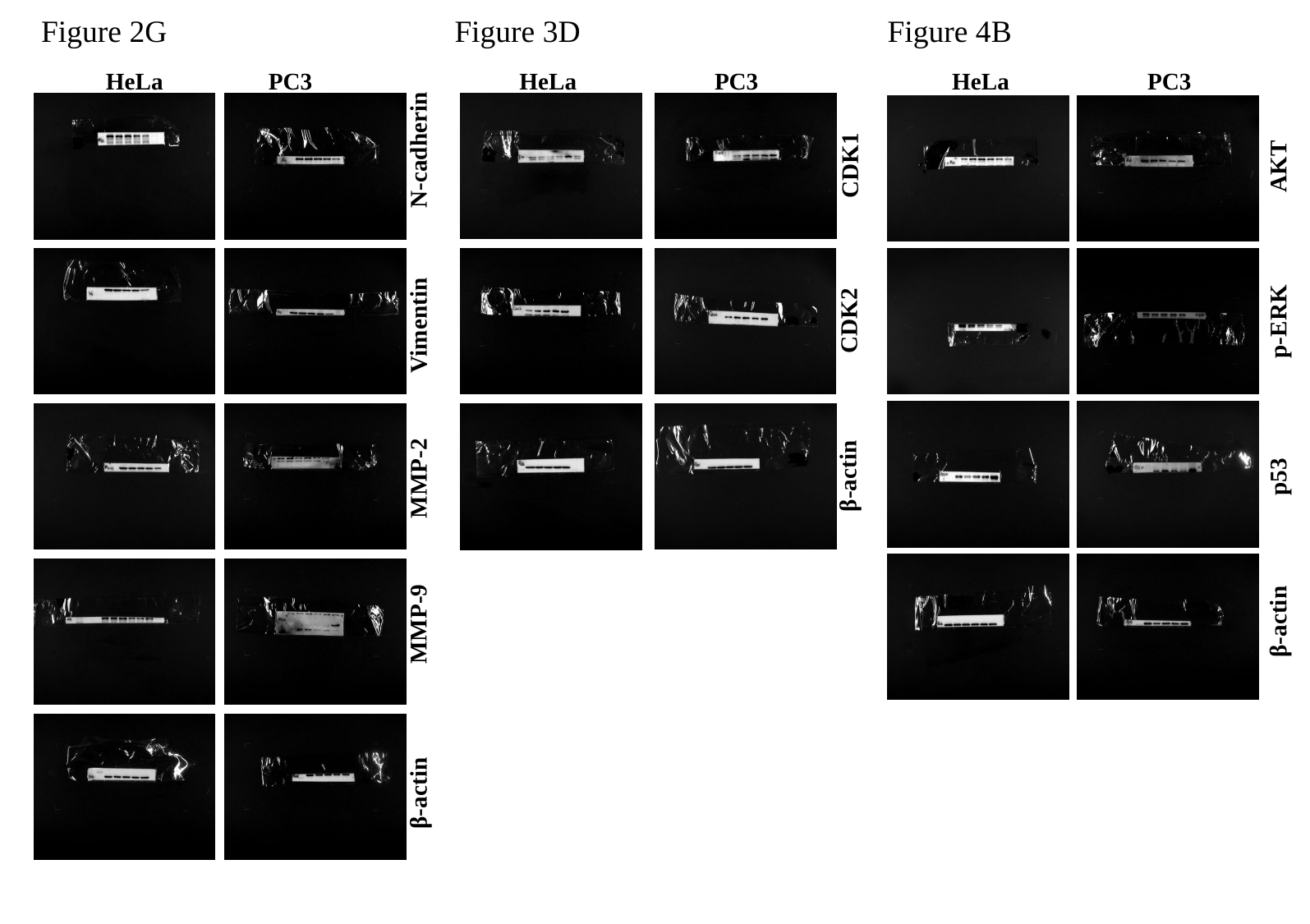

Figure 2G
Figure 3D
Figure 4B
HeLa
PC3
HeLa
PC3
HeLa
PC3
N-cadherin
CDK1
AKT
Vimentin
CDK2
p-ERK
MMP-2
β-actin
p53
MMP-9
β-actin
β-actin
